# Nematicidal Effects of Volatile Organic Compounds from Microorganisms and Plants on Plant-Parasitic Nematodes

**DOI:** 10.3390/microorganisms10061201

**Published:** 2022-06-12

**Authors:** Xiaotong Deng, Xin Wang, Guohong Li

**Affiliations:** State Key Laboratory for Conservation and Utilization of Bio-Resources in Yunnan, Key Laboratory for Southwest Microbial Diversity of the Ministry of Education, Yunnan University, Kunming 650032, China; dxiaotongyn@163.com

**Keywords:** volatile organic compounds, plant-parasitic nematodes, nematicidal, microorganism, plant, toxicity

## Abstract

Plant-parasitic nematodes (PPNs) are one of the most destructive plant pathogens worldwide, and controlling them is extremely challenging. Volatile organic compounds (VOCs), which naturally exist in plants and microorganisms, play an important role in the biological control of PPNs and are considered potential substances for the development of commercial nematicides. This paper summarizes the VOCs produced by microorganisms and plants as well as their toxic effects on PPNs. VOCs from 26 microbial strains and 51 plants that are active against nematodes from over the last decade were reviewed. Furthermore, the mechanisms of toxicity of some VOCs against PPNs are also illustrated.

## 1. Introduction

Nematodes are tiny worms that are some of the most complex and numerous organisms in the world [1]. Nematode species that cause damage to cultivated plant species are called plant-parasitic nematodes (PPNs), and it is difficult to control them [2]. They parasitize a large number of crops all over the world, causing huge economic losses every year. The root-knot nematodes (RKNs), *Meloidogyne* spp., which can attack almost all cultivated plant species, are some of the most economically damaging PPNs on horticultural and field crops [3]. One of the most abundant RKNs in the world is *Meloidogyne incognita*, which is capable of infecting a large number of plants species [4]. *Meloidogyne javanica* is also a highly destructive nematode of *Meloidogyne* spp. It has been reported to be the major pathogen for many crop plants, including tomatoes, potatoes, peanuts, papayas, and rootstocks [5,6,7]. In addition, the pine wood nematode (PWN), *Bursaphelenchus xylophilus*, is the pathogen behind pine wilt disease, which causes serious damage to pine species in many countries [8,9]. In China, *B. xylophilus* is widely spread and causes billions of dollars of economic losses annually [10].

Chemical nematicides are an important part of control strategies. However, they also have significant disadvantages, such as detrimental effects on human health and the environment. With growing public concern about the toxicity of chemical pesticides and ecological protection, the application of many synthetic pesticides has been limited. In recent years, researchers have begun to find and study natural products that can replace typically more effective chemical pesticides [11,12]. Volatile organic compounds (VOCs), which exist in plants and microorganisms, are considered to be potential substances for the development of commercial nematicides [13]. VOCs cover a wide range of alcohols, aldehydes, ketones, esters, ethers, phenols, alkenes, alkynes, and alkyl compounds [14]. Over the past decade, there has been growing awareness of the importance of VOCs.

In this review, we will summarize the nematicidal activity of VOCs produced by microorganisms and plants against PPNs, including *Meloidogyne* spp. and *B. xylophilus*, as well as investigate the nematicidal mechanisms found in some VOCs.

## 2. Nematicidal Effects of Volatile Organic Compounds

### 2.1. Nematicidal Effects of Microbial Volatile Organic Compounds

Many VOCs from different microbial strains have been analyzed to determine their nematicidal activity against *Meloidogyne* spp. and *B. xylophilus* (Table 1).

#### 2.1.1. Nematicidal VOCs from Bacteria

##### Nematicidal VOCs from *Pseudomonas*

VOCs produced by *Pseudomonas putida* 1A00316, isolated from Antarctic soil, have been found to exhibit nematicidal potential against *M. incognita* [15]. From this strain, five compounds (2-octanone, (*Z*)-hexen 1-ol acetate, 2-undecanone, dimethyl disulfide, and 2-nonanone) have shown direct-contact nematicidal activity, with LC_50/48h_ values of 22.712 mg/L, 32.351 mg/L, 22.872 mg/L, 134.330 mg/L, and 63.320 mg/L, respectively. However, only 2-undecanone was found to have fumigant activity against *M. incognita*. Wolfgang et al. reported the nematicidal activity of *Pseudomonas koreensis* T3GI1, *Pseudomonas monteilii* T8GH4, and *Pseudomonas soli* T13GI4 against *M. incognita* and *M. javanica* [16]. The metabolite 1-undecene was found to be one of the main VOCs of *P. monteilii* and *P. soli*, and dimethyl disulfide was found to be the major constituent of all three strains. Furthermore, *P. koreensis* can also produce 3-methoxy-2,5-dimethyl pyrazine, and *P. monteilii* can produce 2-undecanone. Among them, *P. koreensis* was found to have the most significant effect. Although its product, pyrazine, was not as effective as 2-undecanone, it may have an enhancement effect on the nematicidal activity of the total volatiles in *P. koreensis*.

##### Nematicidal VOCs from *Bacillus*

In this study, nematicidal VOCs from *Bacillus* dominated. *Bacillus atrophaeus* GBSC56, derived from plant-growth-promoting rhizobacteria, from the Tibet region of China, was isolated [17]. Among the VOCs produced by GBSC56, dimethyl disulfide, methyl isovalerate, and 2-undecanone exhibited strong nematicidal activity. These three VOCs can induce severe oxidative stress in nematodes, which subsequently caused rapid death. *Bacillus cereus* Bc-cm103 was found to be significantly toxic to *M. incognita* [18]. It can produce nematicidal VOCs, including dimethyl disulfide and S-methyl ester butanethioic acid. *Bacillus megaterium* YMF3.25, as a plant-growth-promoting rhizobacteria, can efficiently and biologically control *M. incognita* via the production of toxic VOCs [19]. Among the VOCs, five compounds, including benzeneacetaldehyde, 2-nonanone, decanal, 2-undecanone, and dimethyl disulphide, showed nematicidal activity against *M. incognita*, and the activity of 2-nonanone and 2-undecanone was even higher. Another plant-growth-promoting bacterium, *Bacillus mycoides* R2, was isolated from tomato rhizosphere soil, which can produce volatile styrene to kill *M. incognita*, and the nematicidal activity of styrene was found to be superior to the nematicides, such as thiacloprid and cadusafos [20].

##### Nematicidal VOCs from Other Bacteria

*Lysinibacillus mangiferahumi* M-GX18^T^, isolated from mango rhizosphere soil, can produce nematicidal VOCs against *M. incognita* [21]. Its active metabolites are 2-octanol, cyclohexene, 3-chloro-4-fluoroben-zaldehyde, dibutyl phthalate, 2-nitro-2-chloropropane, dimethachlore, and dimethyl disulfide. VOCs produced by *Paenibacillus polymyxa* KM2501-1 have exhibited contact nematicidal and fumigant activity against *M. incognita* [22]. Among VOCs with contact nematicidal activity, furfural acetone, 2-undecanol, 4-acetylbenzoic, and 2-decanol acid were the most active, followed by 2-nonanol, 2-undecanone, 2-decanone, and 2-nonanone. Except for 4-acetobenzoic and 2-decanol acid, the other six VOCs had fumigant activity. In addition, furfural acetone has been found to have the highest activity against *M. incognita*, with LC_50/48h_ value of 4.44 mg/L. Xu et al. identified five strains that produce VOCs with nematicidal activity against *M. incognita*, namely, *Pseudochro bactrum saccharolyticum*, *Wautersiella falsenii*, *Proteus hauseri*, *Arthrobacter nicotianae*, and *Achromobacter xylosoxidans* [14]. All five strains can produce dimethyl disulfide and *S*-methyl thiobutyrate. Moreover, *P. saccharolyticum* can produce acetophenone, ethyl 3,3-dimethylacrylate, and nonan-2-one; *W. falsenii* can produce nonan-2-one, 1-methoxy-4-methylbenzene, and butyl isovalerate; *P. hauseri* can produce nonan-2-one and 1-methoxy-4-methylbenzene; *A. nicotianae* can produce acetophenone; and *A. xylosoxidans* can produce acetophenone, nonan-2-one, and 1-methoxy-4-methylbenzene. Among these, *S*-methyl thiobutyrate and nonan-2-one had higher nematicidal activity than the commercially available nematicide dimethyl disulfide. *Virgibacillus dokdonensis* MCCC 1A00493, a deep-sea bacterium, has exhibited significant nematicidal activity against *M. incognita* [23]. Among the VOCs produced by *V. dokdonensis*, acetaldehyde has direct-contact killing, fumigation, and attraction activities, and dimethyl disulfide had direct-contact killing and attraction activities. This indicates that the strain can attract nematodes in order to kill them. Acetaldehyde has been found to be a product of ethanol metabolism in vivo; it has certain toxic effects, and it has been widely reported to be useful as an insect attractant and insecticide [33]. Similar to acetaldehyde, dimethyl disulfide is also an effective component of many insecticides and attractants [34,35]. This compound not only has nematicidal activity against *M. incognita* but also against *B. xylophilus*. Therefore, dimethyl disulfide has been widely studied because of its good field application effect on RKNs and some fungal diseases as well as because of its minor effect on soil microbial communities in the environment. In addition, in another study by Huang et al., the volatile nematicidal compound 4-vinylphenol was identified from *V. dokdonensis* [24].

The nematicidal activity of *Comamonas sediminis* T13GI2 and *Variovorax paradoxus* against *M. incognita* and *M. javanica* was reported [16]. The metabolite 1-undecene was found to be one of the main VOCs of *C. sediminis*, and dimethyl disulfide was found to be the major constituent in the two strains. Yu et al. isolated two marine strains, *Pseudoalteromonas marina* strain H-42 and *Vibrio atlanticus* strain S-16 [30]; their VOCs have strong nematicidal activity against *B. xylophilus*. The main active constituents of *P. marina* H-42 are dimethyl disulphide and dimethyl trisulphide, while for *V. atlanticus* S-16 they are dimethyl disulphid, dimethyl trisulphide, benzaldehyde, and *tert*-butylamine.

#### 2.1.2. Nematicidal VOCs from Fungi

VOCs produced by fungi also have important nematicidal effects. *Duddingtonia flagrans* is a nematode-trapping fungus that produces three-dimensional adhesive networks to trap nematodes, then uses VOCs to kill them [25]. Three VOCs containing cyclohexanol, cyclohexanone, and cyclohexanamine have shown strong nematicidal activity against *M. incognita*. Among these, cyclohexylamine had the highest nematicidal activity, followed by cyclohexanone and cyclohexanol. VOCs produced by *Fusarium oxysporum* strain 21 can kill *M. incognita* [27]. The compounds 2-methylbutyl acetate, 3-methylbutyl acetate, ethyl acetate, and 2-methylpropyl acetate have been identified as having nematicidal activity. Another study showed that the VOC caryophyllene, from *F. oxysporum* 21, may be toxic to *M. incognita* [26].

*Daldinia* cf. *concentrica* is an endophytic fungus that can secrete VOCs with nematicidal activity against *M. javanica* [28]. Interestingly, although the VOCs 3-methyl-1-butanol, (±)-2-methyl-1-butanol, 4-heptanone, and isoamyl acetate each significantly reduces the viability of *M. javanica*, a synthetic volatile mixture composed of these four compounds can completely kill it in a volumetric ratio of 1:1:2:1. The VOCs produced by *Metarhizium brunneum* have nematicidal activity against *Meloidogyne hapla* [29]. The VOCs 1-octen-3-ol and 3-octanone can completely kill *M. hapla*, at the highest doses tested (20 µL). In addition, over the course of this study, we found that 3-octanone appeared to be more toxic than 1-octen-3-ol.

*Annulohypoxylon* sp. FPYF3050 is an endophytic fungus isolated from *Neolitsea pulchella* [31]. The volatile compound produced by this strain, 1,8-Cineole, has strong nematicidal activity, which causes more than 82.9% mortality in *B. xylophilus*. In addition, this compound may synergize with other metabolites of *Annulohypoxylon* sp. FPYF3050 to achieve a stronger nematicidal effect than single compounds. *Trichoderma* is an important biocontrol fungus that produced metabolites that are harmful to nematodes [32]. The volatile compound produced by *Trichoderma* sp. YMF 1.00416, 6-Pentyl-2H-pyran-2-one, has also been demonstrated to have nematicidal activity against *B. xylophilus*. The activity of certain *Trichoderma* metabolites allowing them to be used as biological control agents was attributed, at least in part, to the presence of these active compounds [36].

### 2.2. Nematicidal Effects of Volatile Organic Compounds from Plants

Nematicidal VOCs from different plants have been summarized in Table 2. 

#### 2.2.1. Nematicidal VOCs from Lamiaceae

The essential oil of *Agastache rugosa* has shown strong nematicidal activity against *M. incognita* [37]. The main components of this essential oil are methyleugenol, estragole, and eugenol, and among these three active constituents, both eugenol and methyleugenol have been found to exhibit stronger nematicidal activity against *M. incognita* than estragole. Avato et al. described the nematicidal activities of *Rosmarinus officinalis* and *Thymus satureioides* essential oils [38]. *R. officinalis* essential oil is rich in 1,8-cineole, camphor, and α-pinene. Borneol and thymol play the major roles in *T. satureioides* oil.

Caboni et al. reported nematicidal activity of the essential oils from several mint species against *M. incognita* [39]. The strong nematicidal activity of *Mentha spicata* may be related to the presence of carvone. The nematicidal activity of *Mentha pulegium* against *M. incognita* is attributed to high concentrations of pulegone, a terpene that exhibits considerable activity against *M. incognita*. In addition, menthofuran in *M. pulegium* essential oil also exhibits a certain nematicidal effect. Moreover, the nematicidal effect of *M. pulegium* essential oil on *M. incognita* was also observed in another report [40]. In this report, the nematicidal activities of the essential oils of *Origanum vulgare*, *Origanum dictamnus*, and *Melissa officinalis* were also studied. The high content of carvacrol, a monoterpenoid phenol with many biological activities, may contribute to the strong nematicidal activity of *O. vulgare* and *O. dictamnus* [69,70,71,72]. Carvacrol is also present in *M. pulegium* and *M. officinalis*. Likewise, the oils of *M. pulegium* and *M. officinalis* have been found to contain high pulegone and *trans*-anethole contents, respectively. Moreover, the essential oils of *O. vulgare*, *O. dictamnus*, and *M. officinalis* contain small amounts of terpinen-4-ol. Other compounds have been found in *M. officinalis* oil as well, such as L-carvone geraniol and eugenol. Thymol has been found in *O. dictamnus* and *M. officinalis* oils. All of these volatiles have nematicidal activity, and activity appears to decrease in the following order: L-carvone, pulegone, *trans*-anethole, geraniol, eugenol, carvacrol, thymol, and terpinen-4-ol. Besides carvacrol, thymol is also one of the main components of *O. vulgare*, as reported by Ntalli et al. [1]. In addition, *O. vulgare* has been found to have significant nematicidal activity against *M. javanica* [1]. Besides the plants mentioned above, the essential oil of *Mentha piperita* also has strong nematicidal activity against *M. incognita* [41]. However, the active substances of this plant’s essential oil require further research.

The volatile compound of *Thymus citriodorus* has nematicidal activity against *M. javanica*. Thymol is the main component of *T. citriodorus* [1]. Barbosa et al. reported the nematicidal activity of essential oils extracted from four plants (*Satureja montana*, *Thymbra capitata*, *Thymus caespititius*, and *O. vulgare*) against *B. xylophilus* [42]. Carvacrol, γ-terpinene, and *p*-cymene are the main components of the essential oils of *O. vulgare* and *S. montana*. Carvacrol is also found to be the main component of *T. capitata* and *T. caespititius* oils. 

#### 2.2.2. Nematicidal VOCs from Asteraceae

Nematicidal VOCs are described from *Artemisia herba-alba*. Thujone and camphor are regarded as the major contributors of nematicidal activity against *M. incognita* in *A. herba-alba* [38]. VOCs produced by sunflower (*Helianthus annuus*) seeds cause high mortality in *M. incognita* [43]. VOCs found in the plant, such as 2,3-butanediol, sabinene, eucalyptol, limonene, and α-thujene, have also been reported to be active against *M. incognita* in other studies [53,73,74]. The essential oil of *Artemisia nilagirica* has strong nematicidal activity against *M. incognita* [44].

(*E*)-Ocimenone, the principal component of *Tagetes minuta* essential oil, may play a major role in nematicidal activity against *M. javanica* [45]. Furthermore, another study reported that dihydrotagetone and (*Z*)-β-ocimene, which are also components of the essential oil of *T. minuta*, have certain nematicidal activities against *M. incognita* [46]. The volatile nematicidal component against *M. javanica* of *Eupatorium viscidum* has been found to be 6-methyl-5-hepten-2-one [47]. Sainz et al. studied *Artemisia pedemontana* subsp. *assoana* and showed that its hydrosols have strong nematicidal activity against *M. javanica* [48]. Hydrosols are by-products of distillation and contain highly polar, volatile compounds that dissolve in water and do not persist in essential oils [75]. However, the nematicidal active ingredients in the hydrosols of *A. pedemontana* need to be studied further. 

#### 2.2.3. Nematicidal VOCs from Myrtaceae

The essential oil of *Eucalyptus meliodora* has been found to be very active against *M. incognita* [49]. The main nematicidal compound in *E. meliodora* essential oil is benzaldehyde, with an EC_50/24h_ value of 9 µg/mL. In addition, the activity of *Eucalyptus globulus* essential oil against *M. incognita* have also been reported [41]. D’Addabbo et al. found that *Eucalyptus citriodora* and *Syzygium aromaticum* had strong nematicidal activities against *M. incognita* [50]. The nematicidal activity of *E. citriodora* essential oil is high, which might be attributed to its main component, citronellal. The essential oil of *S. aromaticum* consists almost entirely of eugenol, which may be the main reason for its nematotoxic activity.

#### 2.2.4. Nematicidal VOCs from Brassicaceae

The root of *Armoracia rusticana* is capable of producing allylisothiocyanate, a volatile compound with nematicidal activity against *M. incognita* [51]. Allylisothiocyanate is one of the isothiocyanates that has been found to have high volatility. Isothiocyanates are general biocides, with activity that is based on irreversible interactions with proteins [76]. For the nematicidal activity of volatiles in *Eruca sativa*, erucin, pentyl isothiocyanate, hexyl isothiocyanate, (*E*)-2-hexenal, 2-ethylfuran, and methyl thiocyanate have been found to be the most active against *M. incognita* [52]. VOCs from *Brassica juncea* have been reported to be toxic to *M. incognita* [53], which has been found to yield predominantly sulfur-containing compounds, mainly isothiocyanates. *Nasturtium officinale* leaves can emit VOCs to kill *M. incognita* [54]. In particular, 1-octanol occurring in *N. officinale* shows strong nematicidal activity against *M. incognita*. In addition, other compounds in *N. officinale*, such as 2-methyl-1-propanol and 2-ethyl-1-hexanol, can also induce mortality in *M. incognita*. VOCs produced by broccoli (*Brassica oleracea* var. *italica*) shoots have nematicidal activity against *M. incognita* [43]. Among the VOCs in broccoli, dimethyl disulfide and 3-pentanol have strong nematicidal activities. 

#### 2.2.5. Nematicidal VOCs from Apiaceae 

The essential oil of Cuminum cyminum has been found to have nematicidal activity against both *M. incognita* and *M. javanica* [55]. In essential oils extracted from *C. cyminum* seeds, *p*-cymene and γ-terpinene are the main nematicidal compounds. Ntalli et al. reported the nematicidal activity of the essential oils of Foeniculum vulgare and Pimpinella anisum against *M. incognita* [49]. The essential oils of *F. vulgare* and *P. anisum* both contain *trans*-anethole, estragole, and carvone. Besides, *F. vulgare* is also active against *M. javanica* [56].

#### 2.2.6. Nematicidal VOCs from Rutaceae

The essential oil extracted from Citrus reticulata peel and its main component, limonene, have been shown to be effective against *M. incognita* [57]. The metabolite is also the nematicidal constituent of *Citrus sinensis*, another species of the genus *Citrus* [38]. The nematicidal essential oil of *Ruta graveolens* L. contains 2-undecanone and carvitol. Furthermore, this oil also includes a discrete amount of carvacrol, which may act synergistically with 2-undecanone. More importantly, the chemical is an isomer of thymol, which is highly toxic to *M. incognita* [38]. In another report, it has been noted that 2-nonanone and 2-decanone are also present in *R. graveolens* essential oil, and these two compounds, together with 2-undecanone, are the main components of its insecticidal properties [58].

The essential oils of *Zanthoxylum alatum* has nematicidal activity against *B. xylophilus* [59]. For *Z. alatum* oil, methyl *trans*-cinnamate has the highest nematicidal activity. At a concentration of 0.125–2.0 mg/mL, nematode mortality has been found to be 100%. In addition, the nematicidal activities of linalool, limonene, and 1,8-cineole, which are also the main components in *Z. alatum* oil, have been reported in other studies [77,78,79,80].

#### 2.2.7. Nematicidal VOCs from Other Plants

VOCs from other families, such as Lauraceae, Ericaceae, Amaranthaceae, Poaceae, Anacardiaceae, Geraniaceae, Meliaceae, Capparaceae, Passifloraceae, Lecythidaceae, Piperaceae, Amaryllidaceae, and Simaroubaceae, are also important resources for searching new nematicides.

Eugenol and (*E*)-cinnamaldehyde are the main components against *M. incognita* of *Cinnamomum zeylanicum* (*Cinnamomum verum*) essential oil [50,60]. Eugenol, as 2-alkyl(oxy)phenol, has good activivity against bacteria and insects [81,82]. It has been reported that the allylic double bond and phenolic proton of eugenol are essential for its biological activity [83]. The toxicity of (*E*)-cinnamaldehyde, with respect to nematodes, may be related to its aldehyde structure. It has been reported that (*E*)-cinnamaldehyde is more active as a nematicide than other cinnamic acids within different functional groups [84,85]. 

The main nematicidal component against *M. incognita* in the essential oil of *Rhododendron anthopogonoides* is benzyl acetone [57]. Interestingly, the nematicidal effect of benzyl acetone seemed to be much stronger than the essential oil, and it has even shown activity twice as strong as the oil. The essential oil of *Gaultheria fragrantissima* has been found to have good nematicidal activity against *B. xylophilus* [59]. Methyl salicylate and ethyl salicylate are the most abundant nematicidal ingredients in the essential oil of *G. fragrantissima*. 

*Dysphania ambrosioides* essential oil has a toxic effect on *M. incognita*. The main components of the essential oil extracted from the fruits and seeds of *D. ambrosioides* are (*Z*)-ascaridole, (*E*)-ascaridole, and *p*-cymene [3]. Another study showed that the main components of the essential oil extracted from *D. ambrosioides* are (*Z*)-ascaridole, *p*-cymene, and isoascaridole [62]. All of these compounds have nematicidal activities against *M. incognita*. Among them, (*Z*)-ascaridole has been found to be the most active, with an LC_50_ value of 32.79 µg/mL. Silva et al. screened out two antagonistic species from medicinal plants that are toxic to *M. incognita*, namely, *Cymbopogon nardus* and *D. ambrosioides* [63]. Both plants can emit VOCs that are toxic to *M. incognita*, with citronellal and ascaridole being their major VOCs, respectively. Furthermore, other VOCs from both species, such as 3-methyl-1-butanol, 2-ethylfuran, 2-hexenal, benzaldehyde, α-terpinene, limonene, γ-terpinene, linalool, decanal, citronellol, geraniol, citronellyl acetate, eugenol, dodecanal, etc., have already been shown to have nematicidal activity. The essential oils extracted from *Cymbopogon citratus* has nematicidal activity of against *B. xylophilus* [42]. The essential oils of *C. citratus* are mainly composed of geranial, neral and β-myrcene. 

The essential oils of *Pistacia terebinthus* has strong nematicidal activity against *M. incognita* [49]. The main nematicidal compound in *P. terebinthus* is γ-eudesmol with an EC_50/24h_ value of 50 µg/mL. *Pelargonium asperum* is active against *M. incognita* [50]. The essential oil of *P. asperum* is a complex mixture of terpenes composed of three major components: linalool, citronellol, and geraniol. All of these compounds have a hydroxyl function and are considered to be critical for nematicidal activity.

*Melia azedarach* has attracted much attention due to its range of biological activity against agricultural pests [64]. *M. azedarach* fruits can produce volatile substances with nematicidal activity against *M. incognita*. Organic acids, such as acetic, butyric, and hexanoic, have nematicidal activity against *M. incognita*. However, aldehydes and alcohols have higher nematicidal effects than organic acids. Furfural, 5-hydroxymethylfurfural, and furfurol have shown strong nematicidal activity. Among these compounds, furfural has exhibited the highest nematicidal fumigant activity with an EC_50_ value of 8.52 μg/mL. VOCs from *Azadirachta indica* has been reported to be toxic to *M. incognita* [53]. Alcohols and esters are the main compounds in *A. indica*. 

*Capparis spinosa* can act as an effective natural nematicidal agent against *M. incognita* [65]. Methylisothiocyanate is the main component of *C. spinose*. Moreover, other nematicidal components have also been found in *C. spinose*, such as 2-thiophenecarboxaldehyde and furfural. All three compounds have strong nematicidal effects on *M. incognita*. *Passiflora edulis* seeds can emit VOCs to kill *M. incognita* [54]. The VOCs of Brazil nuts (*Bertholletia excelsa* Bonpl.) and blackpepper (*Piper nigrum* L.) have also been found to inhibit activity by reducing the motility of the nematode, but their active substances have not been reported [66]. 

The volatile component of garlic (*Allium sativum*) that exhibits the most significant nematicidal activity against *M. javanica* is diallyl disulfide, which is a sulfide [67]. The volatile compounds of *Ailanthus altissima* have nematicidal activity against *M. javanica*. The metabolites (*E*,*E*)-2,4-decadienal and (*E*)-2-decenal were found to be the dominant nematicidal components of *A. altissima* [68]. Furthermore, there was a small amount of furfural in the extract. 

## 3. Nematicidal Mechanisms of Volatile Organic Compounds

VOCs kill nematodes via mechanisms that affect the nervous system [86], surface coat, intestine, pharynx, or other tissues [87]. Some compounds, such as eugenol, have been proposed as the primary mode of action with regard to neurotoxicity. Eugenol, a phytochemical belonging to the 2-alkyl(oxy)phenol, has a good toxic effect on nematodes. The compound may act as an acetylcholinesterase inhibitor that blocks octopamine receptors, thus supporting nematicidal effects based on nervous-system targets [88,89]. Carvone is also a potent acetylcholinesterase inhibitor with significant nematicidal activity. This compound is a monoterpenoid with nematicidal potential that is derived from the disruption of nematode nervous systems [90].

Besides the effects on a nematode’s nervous system, volatile nematicidal compounds can also damage other parts of the worm. The exoskeleton and cuticle of nematodes are elastic in order to protect them. The cuticle consists of cross-linked collagens, insoluble proteins called cuticlins, glycoproteins, and lipids. Caboni et al. reported the toxic effects of (*E*,*E*)-2,4-decadienal, (*E*)-2-decenal, and furfural on *M. javanica* [68]. Indeed, aliphatic aldehydes are relatively reactive compounds. Unsaturated aldehydes, such as (*E*,*E*)-2,4-decadienal and furfural, react with the cuticle’s amino or thiol group through a Michael addition. This interaction can cause damage to the cuticle and the leakage of internal fluid, which can cause injury to the nematode. Furthermore, the study showed that, after treatment with 2-nonanone and 2-decanone, the pharyngeal tissue of *M. incognita* shrank or even disappeared, and the intestinal tissue became blurred [22]. It indicated that 2-nonanone and 2-decanone can damage the intestine and pharynx of *M. incognita*.

VOCs also kill nematodes by causing oxidative stress. Oxidative stress causes cell death by inducing damage to DNA, proteins, and lipids [17]. The study showed that methyl isovalerate, 2-undecanone, and dimethyl disulfide have strong nematicidal activity against *M. incognita* [17]. This might be due to the fact that these compounds induce ROS production in nematodes and negatively affect antioxidant enzyme activity in *M. incognita*, resulting in severe oxidative stress and internal damage, thus resulting in the nematode’s rapid death. Kalaiselvi et al. also showed that *A. nilagirica* essential oil may achieve its nematicidal effect by enhancing intracellular ROS production [44].

## 4. Discussion and Conclusions

This study reviewed VOCs produced by 26 microorganism strains and 51 plants as well as their toxic effects on RKNs and PWN that have been economically harmful to plants over the past decade. Bacteria that can produce VOCs with nematicidal activity are mainly concentrated in the genera *Pseudomonas* and *Bacillus*, and active plants include Lamiaceae, Asteraceae, Myrtaceae, Brassicaceae, Apiaceae, Rutaceae, and other plants. In particular, the aromatic plants of the genera *Brassica*, *Agastache*, *Artemisia*, *Rosmarinus*, *Thymus*, *Foeniculum*, *Pimpinella*, *Cymbopogon*, *Mentha*, *Syringa*, *Ruta*, and *Origanum*, as well as the aromatic trees of the genera *Citrus*, *Eucalyptus*, and *Cinnamomum* have been extensively studied.

Interestingly, microorganisms and plants are generally capable of producing their own unique compounds. Only a few compounds exist in both microorganisms and plants, such as dimethyl disulfide, 2-undecanone, 1,8-cineole, benzaldehyde, 2-decanone, and 2-nonanone. Dimethyl disulfide, an active component in many insecticides, has been found to be an effective nematicide and attractant [23]. This compound, which displays strong nematicidal activity against both RKNs and PWN, can be produced by a variety of bacteria and plants. Both dimethyl disulfide and 2-undecanone induce severe oxidative stress in nematodes, resulting in rapid death. 

However, most of these compounds are specific to microorganisms and plants. Among the VOCs produced by microorganisms, many compounds have shown outstanding nematicidal activity against *M. incognita*, such as furfural acetone and 2-decanol. The LC_50/48h_ is 4.44 mg/L for the former and 23.12 mg/L for the latter [22]. These two compounds have been found to have not only contact nematicidal activity and fumigant activity but also certain attractant effects on *M. incognita*. This suggests that these compounds can attract nematodes and then kill them through contact and fumigation.

As plant-specific compounds, furfural, isothiocyanates, carvacrol, and thymol can be produced by a variety of plants, and all show strong nematicidal activity against *Meloidogyne* spp. Ntalli et al. showed that the EC_50_ value of furfural is 8.5 μg/mL [64]. The toxic effects of furfural on nematodes may be attributed to its nucleophilic addition reaction with nematode cuticles, which may lead to the evident cuticle suffering internal damage, followed by the leakage of fluid substances [65]. Isothiocyanates, such as methylisothiocyanate and allylisothiocyanate, have been found to have strong volatility and nematicidal effects. Isothiocyanates are known nematicides that can act as electrophiles to irreversibly interact with proteins, thereby altering protein function [51]. Carvacrol and thymol are the main nematicidal active ingredients in many plants. Both compounds are highly toxic to RKNs and PWN. Lei et al. showed that carvacrol and thymol can interact with a receptor to trigger a signaling cascade that leads to nematode death [42].

VOCs need not act alone; they also have synergism or antagonism between different compounds. Synergism is an effect of different compounds used in combination that is greater than the sum of the individual compounds. Antagonism means a reduction in the biological activity of a mixture of compounds, compared to the activity of each component alone [91]. For example, terpenoids have obvious synergistic effects. According to Ntalli et al., among the essential oils from seven plants indigenous to Greece, the most synergistic terpene pairs are *trans*-anethole/geraniol, *trans*-anethole/eugenol, carvacrol/eugenol, and geraniol/carvacrol [49]. In contrast, besides mixtures with *trans*-anethole, L-carvone mixed with other compounds, such as thymol and eugenol, predominantly exhibits antagonism. In general, the activity of essential oils or the extracts of plants should be related to the content of their main constituents. However, the activity of the mixture is not linearly dependent on the content of the main active components. This may be due to the presence of other compounds in the mixture that have synergism or antagonism with the main components.

All of the VOCs summarized in this paper have the potential to be developed as commercial pesticides. However, further research is needed on their practical application in nematode management and the mechanism of their various toxicities.

## Figures and Tables

**Table 1:** Nematicidal activity of VOCs produced by microorganisms against *Meloidogyne* spp. and *B. xylophilus*.

Nematodes	Microorganisms	VOCs	References
*M. incognita*	*Pseudomonas putida* 1A00316	dimethyl disulfide, 2-nonanone, 2-octanone, (Z)-hexen-1-ol acetate, 2-undecanone	[15]
	*Pseudomonas koreensis* T3GI1	3-methoxy-2,5-dimethyl pyrazine, 1-undecene, dimethyl disulfide	[16]
	*Pseudomonas monteilii* T8GH4	1-undecene, dimethyl disulfide, 2-undecanone	[16]
	*Pseudomonas soli* T13GI4	1-undecene, dimethyl disulfide	[16]
	*Comamonas sediminis* T13GI2	1-undecene, dimethyl disulfide	[16]
	*Variovorax paradoxus* T1GI1	dimethyl disulfide	[16]
	*Bacillus atrophaeus* GBSC56	dimethyl disulfide, methyl isovalerate, 2-undecanone	[17]
	*Bacillus cereus* Bc-cm103	dimethyl disulfide, *S*-methyl ester butanethioic acid	[18]
	*Bacillus megaterium* YFM3.25	benzeneacetaldehyde, 2-nonanone, decanal, 2-undecanone, dimethyl disulphide	[19]
	*Bacillus mycoides* R2	styrene	[20]
	*Lysinibacillus mangiferahumi* M-GX18^T^	2-octanol, cyclohexene, 3-chloro-4-fluoroben-zaldehyde, dibutyl phthalate, 2-nitro-2-chloropropane, dimethachlore, dimethyl disulfide	[21]
	*Paenibacillus polymyxa* KM2501-1	furfural acetone, 2-decanol, 2-undecanone, 2-undecanol 2-decanone, 2-nonanol	[22]
	*Pseudochrobactrum saccharolyticum* AM180484	dimethyl disulfide, *S*-methyl thiobutyrate, acetophenone, ethyl 3,3-dimethylacrylate, nonan-2-one	[14]
	*Proteus hauseri* JN092591	dimethyl disulfide, *S*-methyl thiobutyrate, nonan-2-one, 1-methoxy-4-methylbenzene	[14]
	*Wautersiella falsenii* AM238687	dimethyl disulfide, *S*-methyl thiobutyrate, nonan-2-one, 1-methoxy-4-methylbenzene, butyl isovalerate	[14]
	*Arthrobacter nicotianae* JQ071518	dimethyl disulfide, *S*-methyl thiobutyrate, acetophenone	[14]
	*Achromobacter xylosoxidans* AF411019	dimethyl disulfide, *S*-methyl thiobutyrate, acetophenone, nonan-2-one, 1-methoxy-4-methylbenzene	[14]
	*Virgibacillus dokdonensis MCCC* 1A00493	acetaldehyde, dimethyl disulfide, 4-vinylphenol	[23,24]
	*Duddingtonia flagrans*	cyclohexanamine, cyclohexanone, cyclohexanol	[25]
	*Fusarium oxysporum* 21	2-methylbutyl acetate, 3-methylbutyl acetate, ethyl acetate, 2-methylpropyl acetate, caryophyllene	[26,27]
*Meloidogyne javanica*	*Pseudomonas koreensis* T3GI1	3-methoxy-2,5-dimethyl pyrazine, 1-undecene, dimethyl disulfide	[16]
	*Variovorax paradoxus* T1GI1	dimethyl disulfide	[16]
	*Comamonas sediminis* T13GI2	1-undecene, dimethyl disulfide	[16]
	*Pseudomonas monteilii* T8GH4	1-undecene, dimethyl disulfide, 2-undecanone	[16]
	*Pseudomonas soli* T13GI4	1-undecene, dimethyl disulfide	[16]
	*Daldinia* cf. *concentrica*	3-methyl-1-butanol, (±)-2-methyl-1-butanol, 4-heptanone, isoamyl acetate	[28]
*Meloidogyne hapla*	*Metarhizium brunneum*	1-octen-3-ol, 3-octanone	[29]
*Bursaphelenchus xylophilus*	*Pseudoalteromonas marina* H-42	dimethyl disulphide, dimethyl trisulphide	[30]
	*Vibrio atlanticus* S-16	dimethyl disulphid, dimethyl trisulphide, benzaldehyde, *tert*-butylamine	[30]
	*Annulohypoxylon* sp. FPYF3050	1,8-cineole	[31]
	*Trichoderma* sp. YMF 1.00416	6-pentyl-2H-pyran-2-one	[32]

**Table 2:** VOCs produced by plants against *Meloidogyne* spp. and *B. xylophilus*.

Nematode	Plant Sources	Family	VOCs	References
*M*. *incognita*	*Agastache rugosa*	Lamiaceae	methyleugenol, estragole, eugenol	[37]
*M*. *incognita*	*Rosmarinus officinalis*	Lamiaceae	1,8-cineole, camphor, α-pinene	[38]
*M*. *incognita*	*Thymus satureioides*	Lamiaceae	borneol, thymol	[38]
*M*. *incognita*	*Mentha spicata*	Lamiaceae	carvone	[39]
*M*. *incognita*	*Mentha pulegium*	Lamiaceae	menthofuran, pulegone, *trans*-anethole, carvacrol	[39,40]
*M*. *incognita M.javanica*	*Origanum vulgare*	Lamiaceae	carvacrol, thymol, terpinen-4-ol	[1,40]
*M*. *incognita*	*Origanum dictamnus*	Lamiaceae	carvacrol, thymol, terpinen-4-ol	[40]
*M*. *incognita*	*Melissa officinalis*	Lamiaceae	L-carvone, *trans*-anethole, geraniol, eugenol, carvacrol, thymol, terpinen-4-ol	[40]
*M*. *incognita*	*Mentha piperita*	Lamiaceae	-	[41]
*M. javanica*	*Thymus citriodorus*	Lamiaceae	thymol	[1]
*B. xylophilus*	*Satureja montana*	Lamiaceae	carvacrol, γ-terpinene, *p*-cymene	[42]
*B. xylophilus*	*Thymbra capitata*	Lamiaceae	carvacrol	[42]
*B. xylophilus*	*Thymus caespititius*	Lamiaceae	carvacrol	[42]
*B. xylophilus*	*Origanum vulgare*	Lamiaceae	carvacrol, γ-terpinene, *p*-cymene	[42]
*M*. *incognita*	*Artemisia herba-alba*	Asteraceae	thujone, camphor	[38]
*M*. *incognita*	*Helianthus annuus*	Asteraceae	2,3-butanediol, sabinene, eucalyptol, limonene, α-thujene	[43]
*M*. *incognita*	*Artemisia nilagirica*	Asteraceae	-	[44]
*M*. *incognita M.javanica*	*Tagetes minuta*	Asteraceae	dihydrotagetone, (*Z*)-β-ocimene, (*E*)-ocimenone	[45,46]
*M. javanica*	*Eupatorium viscidum*	Asteraceae	6-methyl-5-hepten-2-one	[47]
*M. javanica*	*Artemisia pedemontana* subsp. *assoana*	Asteraceae	-	[48]
*M*. *incognita*	*Eucalyptus meliodora*	Myrtaceae	*trans*-anethole, benzaldehyde	[49]
*M*. *incognita*	*Eucalyptus globulus*	Myrtaceae	-	[41]
*M*. *incognita*	*Eucalyptus citriodora*	Myrtaceae	citronellal, citronellol, citronellyl formate	[41,50]
*M*. *incognita*	*Syzygium aromaticum*	Myrtaceae	eugenol	[50]
*M*. *incognita*	*Armoracia rusticana*	Brassicaceae	allylisothiocyanate	[51]
*M*. *incognita*	*Eruca sativa*	Brassicaceae	erucin, pentyl isothiocyanate, hexyl isothiocyanate, (*E*)-2-hexenal, 2-ethylfuran, methyl thiocyanate	[52]
*M*. *incognita*	*Brassica juncea*	Brassicaceae	isothiocyanate	[53]
*M*. *incognita*	*Nasturtium officinale*	Brassicaceae	1-octanol	[54]
*M*. *incognita*	Broccoli (*Brassica oleracea* L.)	Brassicaceae	dimethyl disulfide, 3-pentanol	[43]
*M*. *incognita M.javanica*	*Cuminum cyminum*	Apiaceae	γ-terpinene, *p*-cymene	[55]
*M*. *incognita*	*Pimpinella anisum*	Apiaceae	estragole, *trans*-anethole, carvone	[49]
*M*. *incognita*	*Foeniculum vulgare*	Apiaceae	estragole, *trans*-anethole, carvone	[49]
*M. javanica*	*Foeniculum vulgare*	Apiacea	*trans*-anethole, estragole	[56]
*M*. *incognita*	*Citrus reticulata*	Rutaceae	limonene	[57]
*M*. *incognita*	*Citrus sinensis*	Rutaceae	limonene	[38]
*M*. *incognita*	*Ruta graveolens* L.	Rutaceae	2-undecanone, carvitol, 2-nonanone, 2-decanone	[41,50,58]
*B. xylophilus*	*Zanthoxylum alatum*	Rutaceae	linalool, limonene, methyl *trans*-cinnamate, 1,8-cineole	[59]
*M*. *incognita*	*Cinnamomum zeylanicum* (*Cinnamomum verum*)	Lauraceae	(*E*)-cinnamaldehyde, eugenol	[50,60]
*M*. *incognita*	*Rhododendron anthopogonoides*	Ericaceae	benzyl acetone	[61]
*B. xylophilus*	*Gaultheria fragrantissima*	Ericaceae	methyl salicylate, ethyl salicylate	[59]
*M*. *incognita*	*Dysphania ambrosioides*	Amaranthaceae	(*Z*)-ascaridole, (*E*)-ascaridole, *p*-cymene, isoascaridole	[3,62,63]
*M*. *incognita*	*Cymbopogon nardus*	Poaceae	citronellal	[63]
*B. xylophilus*	*Cymbopogon citratus*	Poaceae	geranial, neral, ββ-myrcene	[42]
*M*. *incognita*	*Pistacia terebinthus*	Anacardiaceae	γ-eudesmol	[49]
*M*. *incognita*	*Pelargonium asperum*	Geraniaceae	linalool, citronellol, geraniol	[41,50]
*M*. *incognita*	*Melia azedarach*	Meliaceae	acetic, butyric, hexanoic, furfural, furfurol, 5-hydroxymethylfurfural	[64]
*M*. *incognita*	*Azadirachta indica*	Meliaceae	-	[53]
*M*. *incognita*	*Capparis spinosa*	Capparaceae	methylisothiocyanate, furfural, 2-thiophenecarboxaldehyde	[65]
*M*. *incognita*	*Passiflora edulis*	Passifloraceae	-	[54]
*M*. *incognita*	*Bertholletia excelsa*	Lecythidaceae	-	[66]
*M*. *incognita*	*Piper nigrum* L.	Piperaceae	-	[66]
*M. javanica*	garlic (*Allium sativum*)	Amaryllidaceae	diallyl disulfide	[67]
*M. javanica*	*Ailanthus altissima*	Simaroubaceae	(*E*,*E*)-2,4-decadienal, (*E*)-2-decenal, furfural	[68]

## Data Availability

Not applicable.
